# Inhibition of endosomal fusion activity of influenza virus by *Rheum tanguticum* (da-huang)

**DOI:** 10.1038/srep27768

**Published:** 2016-06-15

**Authors:** Ta-Jen Lin, Chwan-Fwu Lin, Cheng-Hsun Chiu, Ming-Chung Lee, Jim-Tong Horng

**Affiliations:** 1 Department of Biochemistry and Graduate Institute of Biomedical Sciences, College of Medicine, Chang Gung University, 259 Wen-Hwa First Road, Kweishan, Taoyuan 333, Taiwan, R.O.C; 2 Department of Cosmetic Science, Chang Gung University of Science and Technology, Taoyuan 333, Taiwan, R.O.C; 3 Research Center for Industry of Human Ecology, Chang Gung University of Science and Technology, Taoyuan 333, Taiwan, R.O.C; 4 Molecular Infectious Disease Research Center, Chang Gung Memorial Hospital, Kweishan, Taoyuan 333, Taiwan, R.O.C; 5 Brion Research Institute of Taiwan, New Taipei City 231, Taiwan, R.O.C; 6 Research Center for Emerging Viral Infections, Chang Gung University, Kweishan, Taoyuan 333, Taiwan, R.O.C

## Abstract

Rhubarb (*Rheum tanguticum*; da-huang in Chinese medicine) is a herbal medicine that has been used widely for managing fever and removing toxicity. In this study, we investigated how rhubarb inhibits influenza virus during the early stage of the infectious cycle using different functional assays. A non-toxic ethanolic extract of rhubarb (Rex) inhibited several H1N1 subtypes of influenza A viruses in Madin–Darby canine kidney cells, including strains that are clinically resistant to oseltamivir. Time course analysis of Rex addition showed that viral entry was one of the steps that was inhibited by Rex. We also confirmed that Rex effectively inhibited viral attachment and penetration into the host cells. The inhibition of red blood cell haemolysis and cell–cell fusion by Rex suggests that Rex may block haemagglutinin-mediated fusion (virus–endosome fusion) during the fusion/uncoating step. Rex has the capacity to inhibit influenza viruses by blocking viral endocytosis. Thus, rhubarb might provide an alternative therapeutic approach when resistant viruses become more prevalent.

Influenza viruses belong to the Orthomyxoviridae family and they possess negative-sense, single-stranded, and segmented RNA genomes. Influenza A virus strains have been categorized into different subtypes based on their haemagglutinin (HA) and neuraminidase (NA) antigens^1^. At present, 18 different HAs (H1 to H18) and 11 different NAs (N1 to N11) have been identified^2^,^3^. The genetic composition of influenza viruses allows them to evolve via the reassortment of different gene segments to yield highly virulent strains, which can lead to global pandemics^4^.

Influenza A virus binds to N-acetylneuraminic acid on cell surfaces and enters cells via receptor-mediated endocytosis, followed by fusion with early endosomes. The host v-ATPase is activated by an influx of protons, which leads to HA proteolytic cleavage to HA1 and HA2^5^. HA1 is responsible for cell surface receptor binding. In the late endosome, HA2 fusion is activated and the subsequent uncoating step leads to the release of viral ribonucleoprotein (vRNP) complexes into the cytosol. The vRNP complex can bind to the cellular nuclear import machinery via nuclear localization signals to enter the nucleus^6^. vRNP complexes provide a template for both transcription and replication^7^. After replication, viral RNA (vRNA) combines with viral polymerases to form vRNP complexes. Viral matrix protein 1 (M1) and non-structural protein 2 (also known as NS2 or nuclear export protein) facilitate the export of vRNP complexes into the cytoplasm^8^. HA, NA, and vRNP complexes assemble on cell membranes, thereby leading to budding.

The evolution of influenza A viruses is related to their circulation among animals and humans, where pandemic outbreaks with substantial mortality and morbidity have been documented since 1918. The most recent pandemic was caused by the swine-origin influenza A virus (also known as pandemic H1N1 or H1N1pdm), which was detected in humans in 2009. Thus, intervention is needed urgently given the continual outbreaks.

Current anti-influenza virus drug development is focused on interfering with the viral life cycle. Drugs that target the proton channel formed by the viral M2 protein of influenza A virus are used clinically, such as adamantanes (amantadine and rimantadine)^9^. However, prolonged treatment and an immunocompromised status can be conducive to the selection of drug-resistant mutations. Previous studies indicate that influenza strains H3N2 and H1N1pdm are resistant to adamantanes^10^,^11^. NA inhibitors (NAIs) can prevent virus particle budding and release, and orally bioavailable oseltamivir and inhaled zanamivir belong to the NAI class of drugs. These two drugs are currently recommended for the treatment of both influenza A and influenza B viruses. However, recent clinical research has shown that oseltamivir-resistant mutant lines are emerging^12^,^13^. Numerous studies have focused on developing antiviral drugs using natural resources such as traditional herbal medicines. Rhubarb (*Rheum tanguticum*; da-huang in Chinese medicine) is a herbal medicine that has been used to combat microorganisms, inflammation, fever, and viral infection^14^,^15^,^16^. Medical applications are based on the rhubarb rhizome. Rhubarb may suppress DNA viruses like hepatitis B virus and herpes simplex virus^17^,^18^. It is also reported that Rhubarb can inhibit RNA viruses, such as coxsackievirus B3^19^. However, although rhubarb has been used to treat influenza-like fever, no specific antiviral mechanism against influenza virus has been identified. Thus, in the present study, we used various functional assays to determine the stage of the viral life cycle inhibited by rhubarb and we investigated the underlying antiviral mechanism.

## Results

### The ethanolic extract of rhubarb (Rex) has antiviral activity

**Figure 1:**
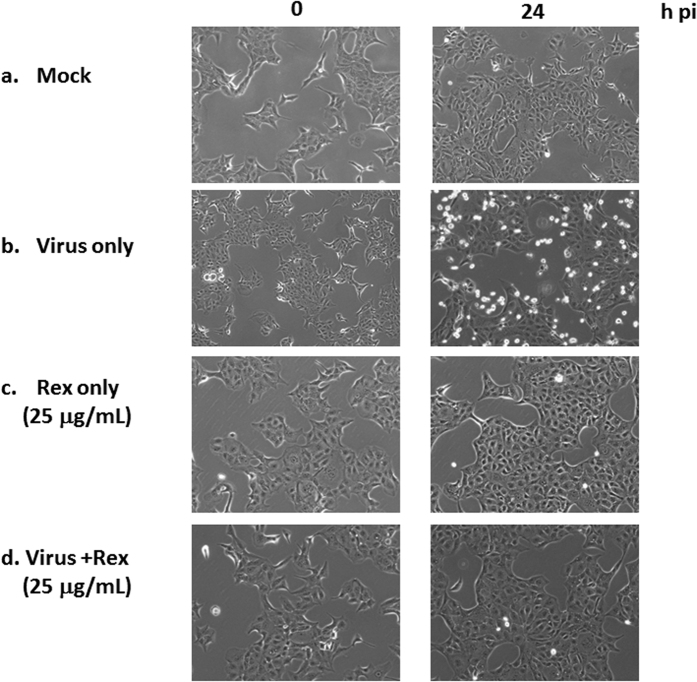
Rex inhibited virus-induced CPE in MDCK cells. Influenza virus-infected MDCK cells were treated with Rex, and virus-induced CPE was recorded at 0 and 24 h pi using a Zeiss Axiovert 200 M microscope. The figure shows a representative result based on three reproducible experiments.

The spectrum of inhibition in Madin–Darby canine kidney (MDCK) cells suggested that Rex inhibited all of the H1N1 subtypes among influenza A virus strains, including H1N1pdm strains and strains that are clinically resistant to oseltamivir (Table 1). Rex effectively suppressed virus-induced cell death with a half-maximal effective concentration (EC_50_) of 11.06 ± 2.19 μg/mL, a 50% cytotoxicity concentration (CC_50_) of 110.71 ± 13.97 μg/mL, and a selectivity index (SI) of approximately 10 (Table 1). However, Rex had no activity against H3N2 subtypes or influenza B viruses. Thus, Rex had more specific effects against the H1N1 subtypes of influenza A virus. We confirmed this Rex-mediated inhibition based on the virus-induced cytopathic effect (CPE) observed by microscopy (Fig. 1). The cells exhibited a bright round shape due to CPE at 24 h post infection (h pi) with the virus (panel b, Fig. 1). The CPE was greatly alleviated when treated with non-toxic 25 μg/mL Rex (panel d, Fig. 1).

### Rex exhibited antiviral activity at different viral replication stages

**Figure 2:**
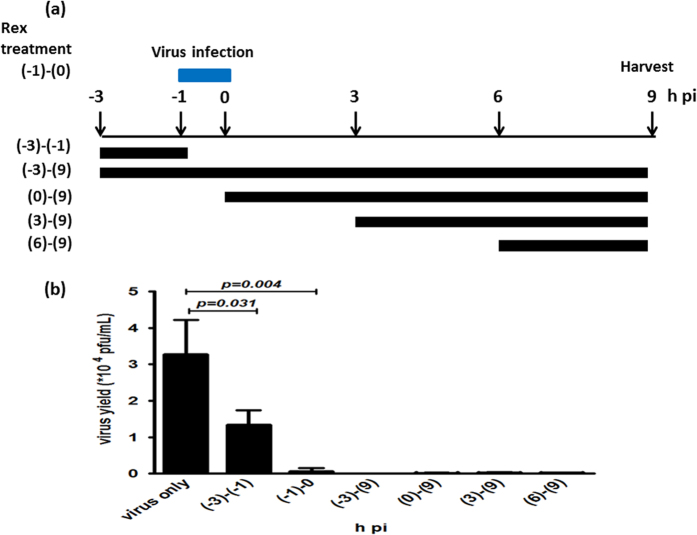
Rex inhibited influenza virus replication at different stages of the life cycle in a time-of-addition assay. (**a**) Schematic diagram of Rex treatment. MDCK cells were infected with influenza virus A/WSN/33 (MOI = 0.1) at −1 to 0 h. Rex was incubated with cells for the durations indicated. All supernatants were collected at 9 h pi to determine the titres using a plaque-forming assay (**b**). The results represent the means ± SD based on three replicates.

In order to explore the antiviral mechanism of Rex, we performed a time-of-addition assay within a single infectious cycle. Influenza virus A/WSN/33 was used to infect MDCK cells at a multiplicity of infection (MOI) of 0.1 based on treatment with Rex (25 μg/mL) at six intervals (Fig. 2a). The results showed that the addition of Rex before virus adsorption partially reduced the virus yields at −3 to −1 h pi, but viral replication was suppressed significantly at the other five treatment times, i.e., at −3 to 9 h, −1 to 0 h, 0 to 9 h, 3 to 9 h, and 6 to 9 h pi (Fig. 2b). This indicates that the activity of Rex against the influenza virus occurred via a variety of different mechanisms, including effects on host cell surface binding, virus entry, genome uncoating, transcription/replication, and virus release. We investigated the mechanisms related to early-stage inhibition by Rex in the present study.

### Inhibition of viral RNA and protein synthesis after adding Rex during viral adsorption

**Figure 3:**
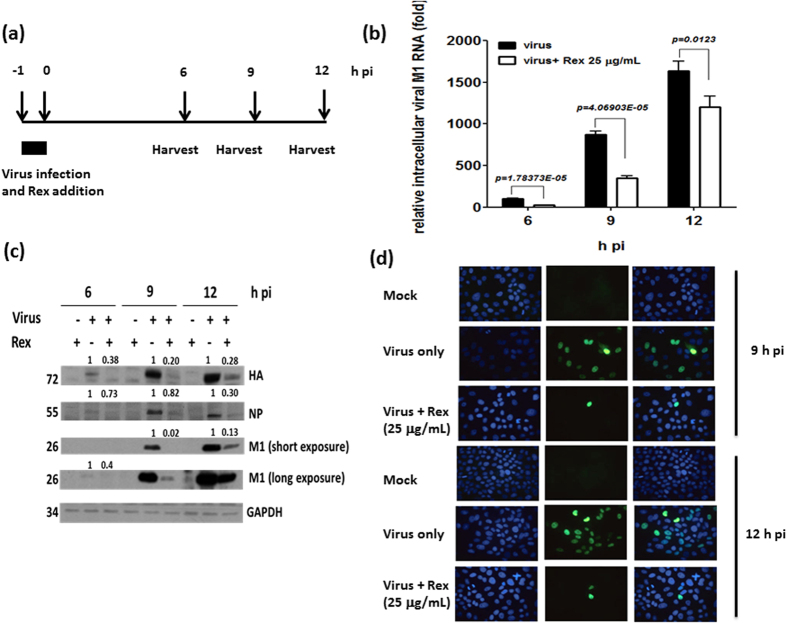
Rex affected influenza viral RNA and protein synthesis. MDCK cells were infected with influenza virus A/WSN/33 (MOI = 0.1) in the presence of Rex (25 μg/mL) at −1 to 0 h. (**a**) Schematic diagram of Rex treatment. The cells were collected for analysis by RT-qPCR (**b**), western blotting (**c**), and immunofluorescence microscopy (**d**). (**b**) viral RNA expression was detected by qPCR using specific primers for *M1*. A representative result is shown based on two reproducible experiments, where each condition was performed in triplicate. (**c**) The viral protein expression levels were determined by immunoblotting. Cell lysates (30 μg/lane) were subjected to SDS-PAGE and western blotting using antibodies against viral proteins (HA, NP, and M1) and an internal control (GAPDH). A representative result is shown based on three experiments. (**d**) Indirect immunofluorescence was evaluated using specific NP antibodies. Cellular nuclei were stained with Hoechst dye. A representative result is shown based on three experiments.

We determined whether the viral RNA and protein levels changed when Rex was added during the viral entry step at −1 to 0 h pi. Influenza virus A/WSN/33 was used for infection and the intracellular RNA and protein lysate were harvested at the times indicated (Fig. 3a). Rex inhibited viral RNA synthesis from 6 h pi according to reverse transcription-quantitative PCR (RT-qPCR) (Fig. 3b). The viral protein levels were markedly reduced at 6, 9, and 12 h pi (Fig. 3c). The subcellular nuclear localization of the viral nucleoprotein (NP) was not perturbed by Rex treatment according to an immunofluorescence microscopic assay (Fig. 3d). However, the viral NP expression level was suppressed greatly at the times indicated post infection, which was consistent with the western immunoblotting data (Fig. 3d). This suggests that Rex may affect virus entry and then interfere with vRNA and protein synthesis.

**Figure 4:**
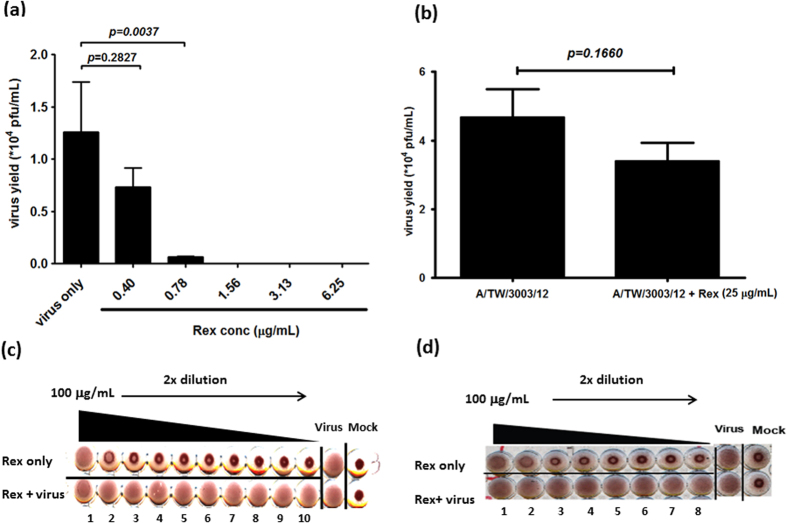
Rex might target viral particles but not receptor binding ability of influenza virus H1N1. (**a,b**) virus binding assay. Influenza virus A/WSN/33 (a) or A/TW/3003/12 (H3N2) (**b**) at an MOI of 0.1 was incubated with Rex (0.40 to 6.25 μg/mL for H1N1 and 25 μg/mL for H3N2) or solvent control (0.1% DMSO) at 4 °C for 60 min. A plaque-forming assay was performed using these viruses. The results represent the means ± SD based on three replicates. (**c,d**) HI assay. A HI assay was performed using guinea pig RBCs. Influenza virus A/WSN/33 (**c**) or A/TW/3003/12 (H3N2) (**d**) of 4X HA was treated with serially diluted Rex for 30 min at room temperature. Subsequently, 0.5% guinea pig RBCs were added and incubated at 4 °C for 60 min. Inhibition of influenza virus-induced RBCs was then measured.

**Figure 5:**
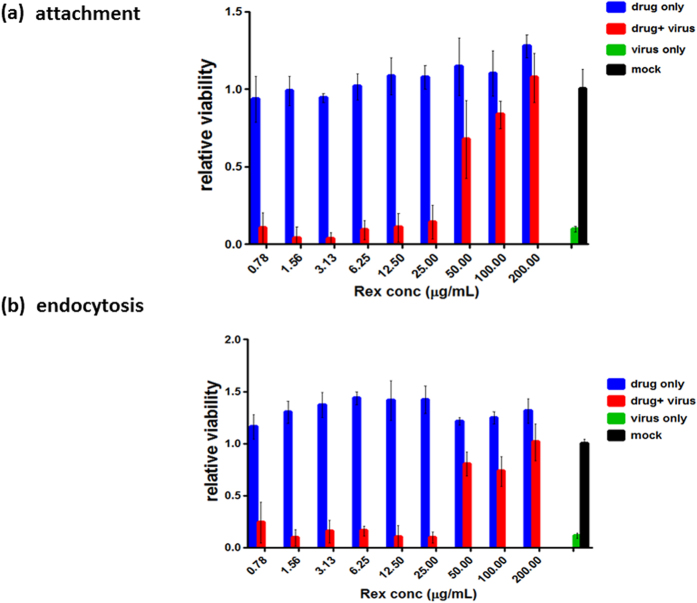
Rex affected viral attachment and penetration into host cells. (**a**) Attachment assay. MDCK cells were incubated with influenza virus A/WSN/33 and serially diluted Rex for 1 h on ice, and the cell viability was then measured using MTT. (**b**) Penetration assay. Influenza virus A/WSN/33 was pre-adsorbed onto MDCK cells for 1 h on ice. The cells were then washed twice with HBSS, and serially diluted Rex was added, before incubating the mixture at 37 °C for 1 h. The mixture was incubated for a further 72 h and the cell viability was determined by an MTT assay. The results represent the means ± SD based on three replicates.

In order to determine whether Rex can target the viral particle directly to inhibit viral entry, we co-incubated influenza virus A/WSN/33 and Rex for 1 h. The mixture was then diluted at least 10-fold in order to obtain non-inhibitory concentrations for determining the viral titre. The results showed that Rex effectively suppressed the viral titre in a dose-dependent manner (Fig. 4a). Next, we tested whether Rex could bind to the non-responsive influenza A/TW/3003/12 (H3N2), against which Rex had no protective effect (Table 1). Although we used a concentration of 25 μg/mL, there was no significant difference in inhibition between the virus alone and the Rex-treated virus (Fig. 4b). These results demonstrate that Rex inhibits influenza viruses by targeting the H1N1 virion but not the virion of H3N2 subtypes. HA is a major membrane glycoprotein and is responsible for cell-surface receptor binding (HA1) and fusion/genome uncoating (HA2) at an early stage of replication. We performed a haemagglutination inhibition (HI) assay to explore whether Rex could specifically suppress HA1 binding activity to red blood cell (RBC) (Fig. 4c,d). The results show that Rex could not reduce influenza virus-induced RBC agglutination (Fig. 4c,d). It is suggested that Rex may not bind to the HA receptor binding region (HA1). We performed viral attachment and penetration assays to elucidate the steps involved in the inhibitory effect of Rex during viral entry. In the attachment inhibition assay, Rex was co-incubated with influenza virus A/WSN/33 during adsorption to MDCK cells on ice. We then measured the cell viability as an indicator of protection by Rex (Fig. 5a). Rex inhibited influenza virus attachment to cells in a dose-dependent manner with a half-inhibitory concentration (IC_50_) of 42.86 ± 1.56 μg/mL. Next, we conducted a penetration inhibition assay to examine HA-mediated endocytosis, which follows attachment. Influenza virus was first attached to the cells at 4 °C. In order to allow endocytosis (penetration) to occur, the cells with viruses bound on their surfaces were incubated at 37 °C in the presence or absence of Rex (Fig. 5b). We found that Rex suppressed the penetration of influenza virus at an IC_50_ of 36.27 ± 4.24 μg/mL (Fig. 5b). By contrast, Rex had no protective effect against penetration by the H3N2 subtype, as demonstrated by influenza A/TW/3003/12 (Supplementary Fig. S1). These results show that Rex may target the viral protein HA to inhibit the virus entry step, which includes both attachment and endocytosis.

### Rex inhibited influenza HA fusion

**Figure 6:**
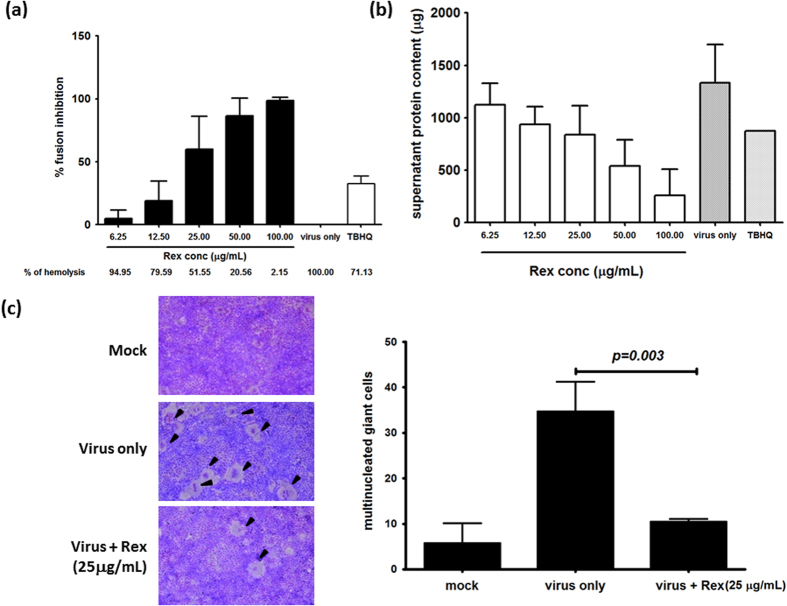
Rex may suppress HA-mediated fusion. (**a,b**) Rex inhibited HA-mediated haemolysis. Influenza virus A/WSN/33 was treated with serially diluted Rex, and guinea pig RBCs were added. Virus-induced haemolysis was measured at 540 nm. The results are shown as the percentage inhibition. Tertiary butylhydroquinone (TBHQ) is a HA-mediated haemolysis inhibitor and it was used as a positive control^36^. (**b**) The total protein content in the supernatants was determined using the Bradford method. (**c**) HA-mediated cell–cell fusion was detected using Giemsa solution, and multinucleated giant cells (shown by arrowheads) were observed by microscopy. The number of multinucleated giant cells was counted in 10 randomly selected fields for each treatment. The results represent the means ± SD based on three independent experiments.

HA is a major membrane glycoprotein that is responsible for endosomal fusion to release viral genomes during the early stages of replication. We performed a RBC haemolysis inhibition assay to determine whether Rex could inhibit HA-mediated fusion. The degree of HA-mediated cell–cell fusion was determined by detecting the haemoglobin content based on the absorbance of the supernatant at 540 nm. We found that RBC haemolysis was suppressed by Rex addition in a dose-dependent manner, with an IC_50_ of about 25 μg/mL (Fig. 6a). We also confirmed this result by measuring the total protein content in the supernatant. The dose of Rex had a linear negative effect on the protein content in the supernatant (Fig. 6b). The RBC haemolysis inhibition assay also suggested that Rex had no protective effect against influenza A/TW/3003/12 (Supplementary Fig. S2).

A cell–cell fusion inhibition assay was used to determine whether Rex inhibits fusion. Influenza virus infection induced HA-mediated multinucleated giant cells (arrowheads, Fig. 6c), but the number of multinucleated giant cells was reduced significantly by treatment with Rex (Fig. 6c). Thus, our results suggest that Rex inhibits influenza virus during the early stage of endocytosis by suppressing HA fusion.

We used four-week-old SPF BALB/C mice for *in vivo* experiments (Fig. S3). We gave Rex twice a day (200 mg/kg/day) from day -7 post infection (d pi). We then challenged mice at 0 d pi with A/WSN/33 by nasal administration. Results showed that body weight was recovered when treated with Rex. We found that Rex protected mice from virus-induced death but this protection was not statistically significant using a Gehan–Breslow–Wilcoxon test.

## Discussion

**Figure 7:**
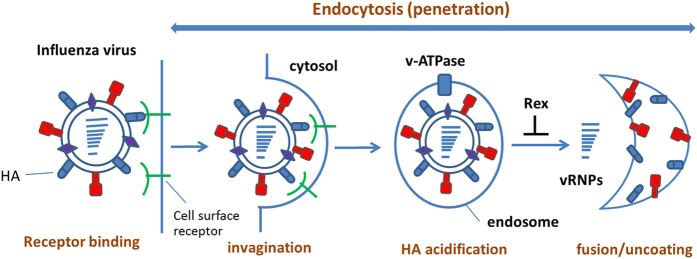
Schematic diagram illustrating Rex-mediated inhibition of viral entry.

In this study, we used a cell model to investigate how Rex suppresses influenza virus replication. Rex had a specific inhibitory effect against H1N1, including oseltamivir-resistant strains (Table 1). Thus, rhubarb might provide an alternative therapeutic approach when resistant viruses become more prevalent. The results of the time-of-addition assay indicated that Rex inhibited different stages of the influenza virus infectious cycle (Fig. 2). Thus, we hypothesized that Rex may interfere with virus entry or other steps, such as vRNP complex export, HA maturation, assembly, and budding. We focused on the early stage of inhibition and found that Rex may target HA-related functions, according to cell-based functional assays. In particular, viral RNA transcription and protein synthesis were suppressed by Rex (Fig. 3) because Rex inhibited virus entry, thereby reducing viral replication. The early steps include receptor binding followed by endocytosis, including HA acidification, fusion, and genome uncoating (Fig. 7). We demonstrated that Rex suppresses membrane fusion to inhibit viral endocytosis, thereby suggesting that the fusion activity mediated by HA was inhibited by Rex (Fig. 6).

The major components of rhubarb are anthraquinone-based compounds. Previous studies indicate that anthraquinone may act as a protein photocleaver under ultraviolet (UV) irradiation and that it has potential as an influenza A virus inhibitor via NA cleavage^20^,^21^. The main bioactive anthraquinone-based derivatives of rhubarb include emodin, aloe-emodin, rhein, chrysophanol, and physcion^22^. These components were confirmed by high-performance liquid chromatography (HPLC, Supplementary Fig. S4). Emodin is a well-known antiviral compound with broad-spectrum efficacy^18^,^23^,^24^,^25^. Aloe-emodin has been reported to suppress the host immune response to influenza A virus via galectin-3^26^. However, when we tested all five of these compounds independently as pure substances, including aloe-emodin, we detected no significant effect against influenza A/WSN (Supplementary Table S1), which may be attributable to the different virus strains used in the assay because Rex has a narrow inhibitory spectrum (Table 1).

HA is crucial for influenza virus during the entry step in the infectious cycle. Influenza HA attaches to a sialic acid receptor on the surface of the host cell within the globular head. The next step involving HA is acid-triggered virus–endosome fusion, which is important for uncoating^27^. The HA fusion peptide at the stalk undergoes a conformational change in the late endosome, where the well-known host factors v-ATPase, Rab7, and LAMP1 participate in this process^14^,^28^. We found that Rex is more specific for the H1N1 subtypes of influenza A virus (Table 1). Therefore, we postulate that Rex targets the H1N1 virus in the entry step. The binding assay showed that a low concentration of Rex was still effective against influenza virus H1N1 replication. However, no protective effect was obtained against influenza virus H3N2, even at a high concentration (Fig. 4). Thus, Rex may directly target the HA to block attachment as well as the fusion and uncoating process^29^. In summary, our results showed that Rex is more specific for the H1N1 subtypes of influenza A virus by inhibiting the entry step through HA stalk targeting to inhibit virus–endosome fusion.

## Methods

### Cell culture and viruses

MDCK cells were grown in DMEM supplemented with 2 mM of l-glutamine, 0.1 mM of non-essential amino acid mixture, antibiotics (100 U/mL penicillin and 0.1 mg/mL streptomycin), and 10% FBS (Biological Industries, Israel). All cells were cultured as monolayers at 37 °C in a humidified incubator under a 5% CO_2_ atmosphere. Influenza virus A/WSN/33 (H1N1) stocks were purchased from the American Type Culture Collection (Manassas, VA, USA) and propagated in MDCK cells. H3N2, H1N1pdm, and the strains with clinical resistance to oseltamivir listed in Table 1 were obtained from the Clinical Virology Laboratory at Chang Gung Memorial Hospital, Taiwan^30^.

### Preparation of Rex

Dried *Rheum tanguticum* Maxim. ex Balf was obtained from Sun Ten Pharmaceutical Co. (New Taipei City, Taiwan) and identified by Brion Research Institute of Taiwan (New Taipei City, Taiwan). The dried rhizome and roots of *Rheum tanguticum* were extracted with 95% ethanol at 50 °C for 4 h. The ethanol was evaporated and the dried solids were then dissolved in DMSO to prepare a 200 mg/mL stock solution of Rex. The Rex solution was stored in small aliquots and kept at −20 °C. The concentrations of the marker compounds in Rex, i.e., aloe-emodin, rhein, emodin, chrysophanol, physcion, sennoside A, and sennoside B, were determined by HPLC-photodiode array method, where a Cosmosil 5C_18_-MS-II column was used as the stationary phase and a gradient comprising phosphoric acid, acetonitrile, and water was used as the eluent. The UV detection wavelength was set at 270 nm. The HPLC results are shown in Supplementary Fig. S4. A voucher specimen of the rhubarb used to prepare Rex in the present study was deposited at the herbarium of Chang Gung University, Taoyuan, Taiwan.

### EC_50_ assay

The EC_50_ was determined as described previously^31^. Briefly, cells were infected with A/WSN/33 virus at 9× TCID_50_ (50% tissue culture infective dose) in the presence of serial dilutions of Rex. After 72 h, the cells were fixed with 4% PFA and stained with crystal violet. The cell density was measured using a VICTOR^3^ Multilabel Plate Reader (PerkinElmer, USA). The EC_50_ was defined as the concentration that caused 50% inhibition of a virus-induced CPE^32^.

### Cytotoxicity assay

The cytotoxicity assay was performed as described previously^31^. Briefly, MDCK cells in 96-well plates were treated with serial dilutions of Rex and then incubated at 37 °C for 3 days. We added 3-(4,5-dimethylthiazol-2-yl)-2,5-diphenyltetrazolium bromide (MTT) (Molecular Probes, USA) in order to determine the cytotoxicity. The absorbance was measured at 570 nm using a VICTOR^3^ Multilabel Plate Reader. The CC_50_ of Rex was defined as the induction of 50% cell death.

### Microscopic examination of virus-induced CPE

MDCK cells in six-well plates were infected with influenza virus A/WSN/33 (MOI = 0.1) for 1 h and the unbound viruses were washed away with PBS. Rex (25 μg/mL) was added and CPE was evaluated at 0 and 24 h pi using a Zeiss Axiovert 200 M microscope with a 10× objective lens.

### Time-of-addition assay

MDCK cells in six-well plates were infected with influenza virus A/WSN/33 (MOI = 0.1) between −1 and 0 h for 1 h. The cells were then treated with Rex (25 μg/mL) at six different intervals: −3 to −1 h (pretreatment), −1 to 0 h (virus absorption), −3 to 9 h, 0 to 9 h, 3 to 9 h, and 6 to 9 h pi. The supernatants were harvested at 9 h pi to determine the viral titre using a plaque-forming assay.

### RNA isolation and RT-qPCR

MDCK cells were infected with influenza virus A/WSN/33 (MOI = 0.1) for 1 h and co-incubated with Rex (25 μg/mL). Cells were harvested at 0, 6, 9, and 12 h pi and the total intracellular RNA was extracted using TRIzol reagent (Invitrogen, USA). The extracted RNA was treated with RQ1 DNase (Promega, USA) in order to remove any DNA contamination. The first strand of cDNA was obtained using M-MLV reverse transcriptase (Invitrogen, USA). The primer sequences for *M1* (A/WSN/33) were 5′‐GAC CAA TCC TGT CAC CTC-3′ (forward) and 5′‐GAT CTC CGT TCC CAT TAA GAG‐3′ (reverse). The *GAPDH* primers were 5′‐AAG AAG GTG GTG AAG CAG GC‐3′ (forward) and 5′‐TCC ACC ACC CTG TTG CTG TA‐3′ (reverse). qPCR was performed using an ABI StepOnePlus sequence detection system (Applied Biosystems, USA). To quantify changes in gene expression, the ΔCt method was used to calculate the relative changes normalized against *GAPDH*, where Ct was defined as the cycle where the fluorescence was significantly greater than the background. The ratio of viral RNA to the internal control was normalized against the 0 h pi control level, which was set arbitrarily to 1.0.

### Western immunoblotting assay

MDCK cells were infected with influenza virus A/WSN/33 (MOI = 0.1) for 1 h and then co-incubated with Rex (25 μg/mL). Cells were harvested at 6, 9, and 12 h pi. The lysates were quantified using the Bradford method and analysed by 10% SDS-polyacrylamide gel electrophoresis (SDS-PAGE) and western immunoblotting. Mouse monoclonal antibodies against influenza virus NP (Abcam, UK), goat anti-M1 (ViroStat, USA), rabbit anti-HA (Genetex Inc., USA), and anti-GAPDH (Santa Cruz Biotechnology, USA) were used for detection. HRP-conjugated secondary antibodies were added. Visible bands were detected using an enhanced chemiluminescence method (Millipore, USA).

### Indirect immunofluorescence microscopy

MDCK cells were seeded at 2 × 10^5^ cells per well onto cover slips and incubated overnight at 37 °C with 5% CO_2_ for 16 h. Cells were infected with influenza virus A/WSN/33 (MOI = 0.1) for 1 h and co-incubated with Rex (25 μg/mL). Cells were fixed with 4% PFA at 9 and 12 h pi for 1 h at room temperature. The cells were permeabilized with 0.5% Triton X-100 in PBS for 3 min and blocked with 0.5% BSA in PBS for 1 h. The cells were then stained with anti-NP antibodies (Abcam, UK) for 1 h and incubated with the appropriate Alexa Fluor 488-labelled secondary antibody (Invitrogen, USA). Cellular nuclei were stained with Hoechst dye (Sigma Aldrich, USA). Fluorescence was detected using a Zeiss Axiovert 200 M microscope (Carl Zeiss Light Microscopy, Germany) with a 100× oil-immersion objective lens.

### Influenza virus–Rex binding assay

The interaction between Rex and the viral particles was analysed using a binding assay. Influenza virus A/WSN/33 or A/TW/3003/12 (H3N2) (MOI = 0.1) was pre-incubated with Rex (0.40 to 6.25 μg/mL at two times serial dilutions) or the solvent control (0.1% DMSO) at 4 °C for 1 h in a rocking incubator. The Rex-treated virus was then used to infect MDCK cells and the viral titre was determined using a plaque-forming assay.

### Haemagglutination inhibition (HI) assay

The HI assay was based on a previously described method^33^. Virus-induced RBC haemagglutination was optically observed and 1 × HAv units was defined as the minimum virus loading required to induce haemagglutination. We chose 4 × HA of influenza virus A/WSN/33 or influenza A/TW/3003/12 for HI assays and the virus was co-incubated with serially diluted Rex for 1 h at room temperature. Two volumes of 0.5% guinea pig RBCs were added for 1 h on ice. Haemagglutination inhibition by Rex was then determined.

### Attachment and penetration assay

In the attachment assay, MDCK cells were pre-adsorbed with 3× TCID_50_ of influenza virus A/WSN/33 in the presence of serially diluted Rex for 1 h on ice. In the penetration assay, MDCK cells were pre-chilled on ice for 30 min and then infected with 3× TCID_50_ of influenza virus A/WSN/33 for 1 h on ice. The cells were washed twice with Hank’s balanced salt solution (HBSS) and incubated with serially diluted Rex at 37 °C for 1 h. Unpenetrated viruses were inactivated with HBSS (pH 2) for 1 min and then neutralized with HBSS (pH 11). After incubation for 72 h, the attachment or penetration inhibition levels were determined based on the cell viability using an MTT assay^31^.

### RBC haemolysis inhibition assay

The RBC haemolysis inhibition assay was modified from a previously described protocol^34^. First, 100 μL of influenza viruses (10^6^ plaque-forming units (pfu)/mL) were pre-treated with 100 μL of serially diluted Rex for 30 min at room temperature. Next, 200 μL of 2% guinea pig RBCs were incubated with this mixture for 30 min with gentle shaking. In order to induce HA-mediated RBC haemolysis, 100 μL of 0.5 M sodium citrate (pH 5.2) was incubated with the reaction mixture with intermittent shaking (60 rpm) for 1 h. The supernatant was collected by centrifugation at 100 × *g* for 5 min and 300 μL of the reaction mixture was used to determine the released haemoglobin by measuring the absorbance with a VICTOR^3^ Multilabel Plate Reader at 540 nm. The fusion inhibition percentage was calculated according to a previously described protocol^34^.

### Cell–cell fusion inhibition assay

The cell–cell fusion inhibition assay was modified from a previously described protocol^35^. MDCK cells were infected with influenza virus A/WSN/33 or A/TW/3003/12 (MOI = 5). Trypsin was added at 9 h pi for 30 min to facilitate cell-surface HA cleavage. The cells were then washed with PBS (pH 7.4) and incubated with Rex (25 μg/mL) for 30 min. The cells were washed twice with PBS before acidic PBS (pH 5.2) treatment for 2 min with gentle shaking at room temperature in order to initiate cell–cell fusion. The cells were washed, incubated at 37 °C for 3 h, and fixed with methanol for 30 s, before staining with 20% Giemsa mixture (Merck Millipore, Germany) for 30 min in the dark at room temperature. The multinucleated giant cells obtained from cell–cell fusion were counted using a Nikon Eclipse TS100 microscope (Nikon Instruments Inc., USA) equipped with a 10× objective lens.

### Ethics

All animal methods and care described in Supplemental Information were carried out in accordance with national guide. They were approved by the Institutional Animal Care and Use Committee of Chang Gung University.

### Data analysis

The data obtained were analysed using GraphPad Prism (version 5.0). The data were expressed as means ± SD. Significant differences were determined using a two-tailed Student’s *t*-test, where *P* < 0.05 was considered significant when comparing two groups. Multiple group comparisons were tested by applying the ANOVA test. The immunoblotting band intensities were quantified using ImageJ and normalized against that of GAPDH.

## Additional Information

**How to cite this article**: Lin, T.-J. *et al*. Inhibition of endosomal fusion activity of influenza virus by *Rheum tanguticum* (da-huang). *Sci. Rep.* **6**, 27768; doi: 10.1038/srep27768 (2016).

## Supplementary Material

Supplementary Information

## Figures and Tables

**Table 1:** Spectrum of inhibition of Rex against different influenza viruses.

Cell line or virus strain	Rhubarb crude extract (μg/mL)		
	CC_50_^a^	EC_50_^b^	SI^e^
Cytotoxic effect			
MDCK	110.71 ± 13.97		
Influenza virus			
A/WSN/33 (H1N1)		11.06 ± 2.19	10
A/TW/90167/09 (H1N1pdm)^c^		2.90 ± 0.96	38
A/TW/90206/09 (H1N1pdm)^c^		7.33 ± 0.75	15
A/TW/7717/09 (H1N1)^d^		3.14 ± 2.11	35
A/TW/7855/09 (H1N1)^d^		5.85 ± 3.85	18
A/TW/6663/09 (H1N1)^d^		8.07 ± 1.75	16
A/TW/3003/12 (H3N2)		>100	–
A/TW/3446/02 (H3N2)		>100	–
B/TW/70325/05		>100	−
B/TW/99/07		>100	−

^a^CC_50_ was determined by MTT assay.

^b^EC_50_ was determined by anti-CPE assay using crystal violet staining.

^c^Pandemic H1N1 (SOIV) strains.

^d^Strains clinically resistant to oseltamivir.

^e^Ratio of CC_50_ to EC_50_.
