# Supplementary material for: Inhibition of endosomal fusion activity of influenza virus by Rheum tanguticum (da-huang)
Source: Sci Rep. 2016 Jun 15;6:27768. doi: 10.1038/srep27768 (PMC4908592; doi:10.1038/srep27768)
Supplement: Supplementary Information [file srep27768-s1.pdf]

Inhibition of endosomal fusion activity of influenza virus by *Rheum tanguticum* (da-huang)

Ta-Jen Lin[1], Chwan-Fwu Lin[2,3], Cheng-Hsun Chiu[4], Ming-Chung Lee[5], Jim-Tong Horng[1,3,4,6,*]

[1] Department of Biochemistry and Graduate Institute of Biomedical Sciences, College of Medicine, Chang Gung University, 259 Wen-Hwa First Road, Kweishan, Taoyuan333, Taiwan, R.O.C.
[2] Department of Cosmetic Science, Chang Gung University of Science and Technology, Taoyuan 333, Taiwan, R.O.C.
[3] Research Center for Industry of Human Ecology, Chang Gung University of Science and Technology, Taoyuan 333, Taiwan, R.O.C.
[4] Molecular Infectious Disease Research Center, Chang Gung Memorial Hospital, Kweishan, Taoyuan333, Taiwan, R.O.C.
[5] Brion Research Institute of Taiwan, New Taipei City 231, Taiwan, R.O.C.
[6] Research Center for Emerging Viral Infections, Chang Gung University, Kweishan, Taoyuan333, Taiwan, R.O.C.

Correspondence and requests for materials should be addressed to J.-T. H.

(email:jimtong@mail.cgu.edu.tw)

Supplemental methods

Ethics. All animal methods and care described in Supplemental Information were carried out in accordance with national guide. They were approved by the Institutional Animal Care and Use Committee of Chang Gung University.

Mouse experiment. Female BALB/C mice (four weeks old) were purchased (BioLASCO Taiwan Co., Ltd.) for *in vivo* experiments. Mice were infected with A/WSN/33 (2×10^5 pfu, $2 \times LD_{50}$) by the nasal route. Rex was prepared at the indicated concentration in 100 μL aqueous solution (containing 5% DMSO) and was administered intraperitoneally twice a day from day -7. Body weight and survival was recorded every day. Survival rate was analysed using the Kaplan–Meier method and statistical significance was determined by the Gehan–Breslow–Wilcoxon test.

Neuraminidase activity inhibition assay. Experiment was performed on a black 96 well plate. MES buffer (32.5 mM MES, 4 mM $CaCl_2$, pH 6.5) was used for virus and drug dilution. Influenza virus A/WSN/33 was co-incubated with serial-diluted Rex at 37°C for 30 min. 50 μM 2-(4-Methylumbelliferyl)-a-D-N-acetylneuraminic acid (MUNANA) was added as neuraminidase substrate at 37 ^{o}C for 60 min. The reaction was stopped with a stop solution (0.1 M glycine buffer containing 25% ethanol, pH 10.7). The absorbance was detected with the excitation wavelength at 360 nM and the emission wavelength at 460 nM by VICTOR^3 Multilabel Plate Reader.

Supplementary Table S1 anti-influenza activity of Rex major anthraquinone-based components.

Rex major anthraquinone-based component	CC_{50}[a] (μM)	EC_{50}[b] (μM)
Aloe-emodin	> 20	> 20
Crysophanic acid	10	> 20
Emodin	> 20	> 20
Physcion	> 20	> 20
Rhein	> 20	> 20
Mixture (a:c:e:p:r = 1:1:1:1:1)	10	2.84 ± 0.91

a, CC_{50} was determined by MTT assay.

b, EC_{50} was used with A/WSN/33 (H1N1) and determined by neutralization assay using crystal violet staining.

a

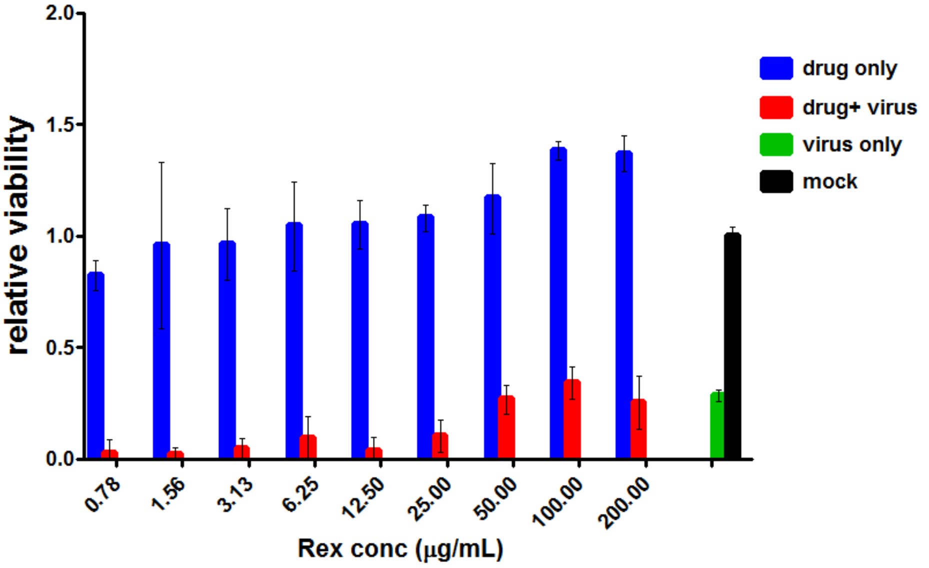

b

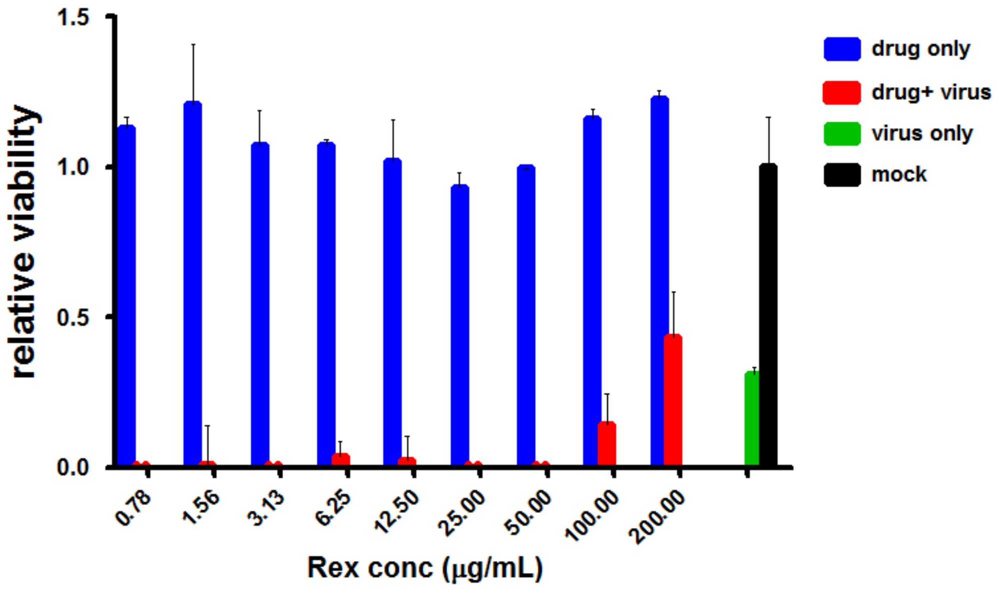

Supplementary Figure S1. Attachment and penetration assays showed that Rex did not suppress A/TW/3003/12 (H3N2) during the early stage.

a

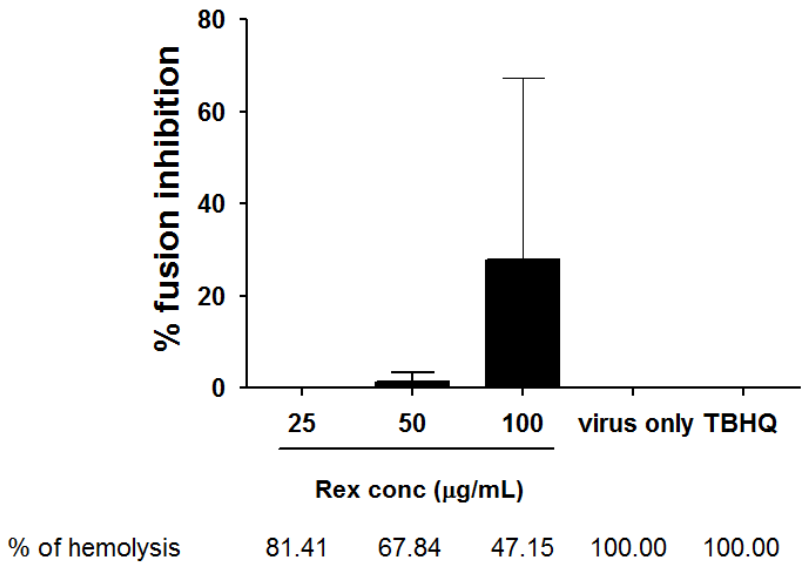

b

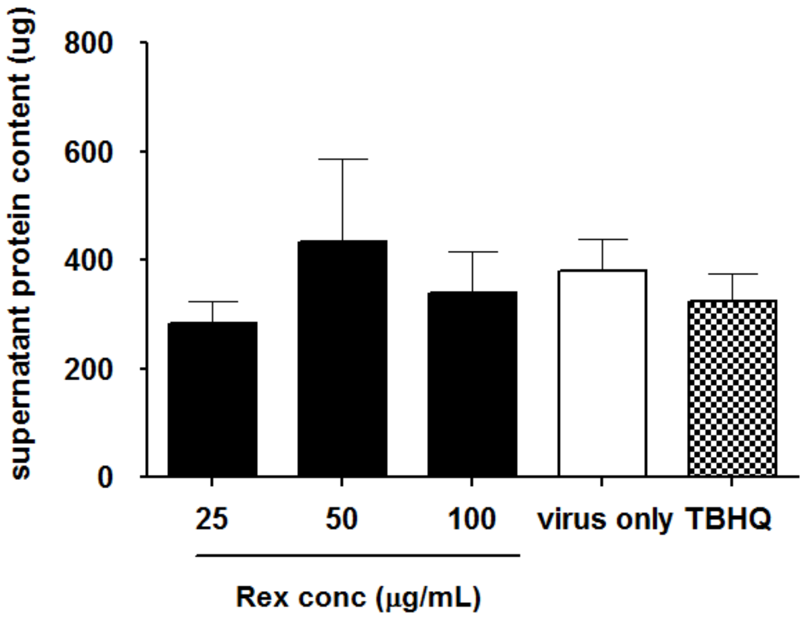

Supplementary Figure S2. Rex did not suppress the fusion activity of A/TW/3003/12 (H3N2).

a

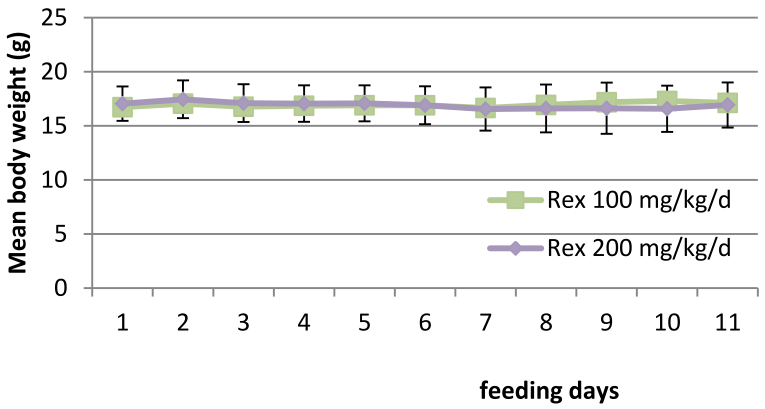

b

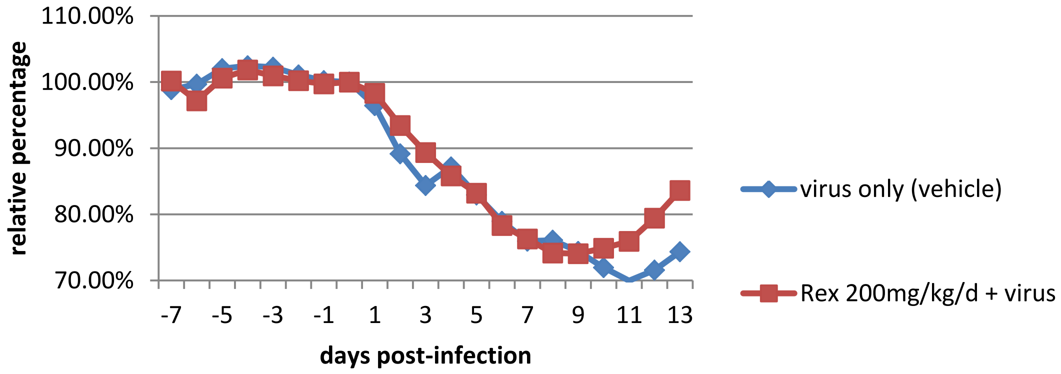

c

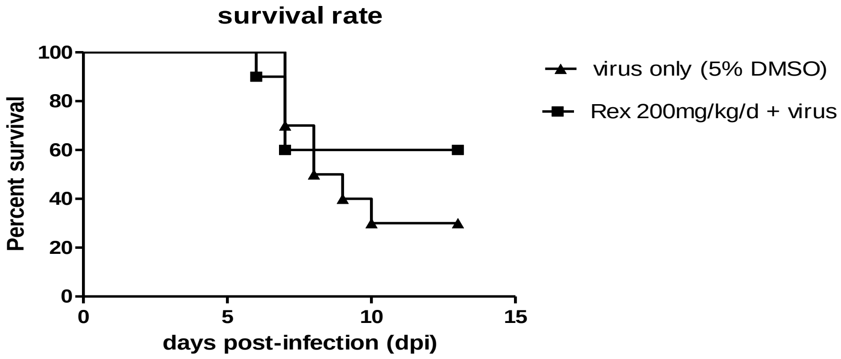

Gehan-Breslow-Wilcoxon Test	
Chi square	0.1948
df	1
P value	0.6589
P value summary	ns
Are the survival curves sig different?	No

Supplementary Figure S3. Rex protective activity *in vivo*. a. Rex toxicity test in BALB/C mice. b. Body weight recovery during Rex treatment. c. Survival rate evaluation for Rex antiviral activity *in vivo*.

(a) Qualitative analysis for aloe-emodin, rhein, emodin, chrysophanol, and physcion detection.

HPLC conditions

Detection wavelength	270 nM
Flow rate	0.8 mL/min
Run time	55 min

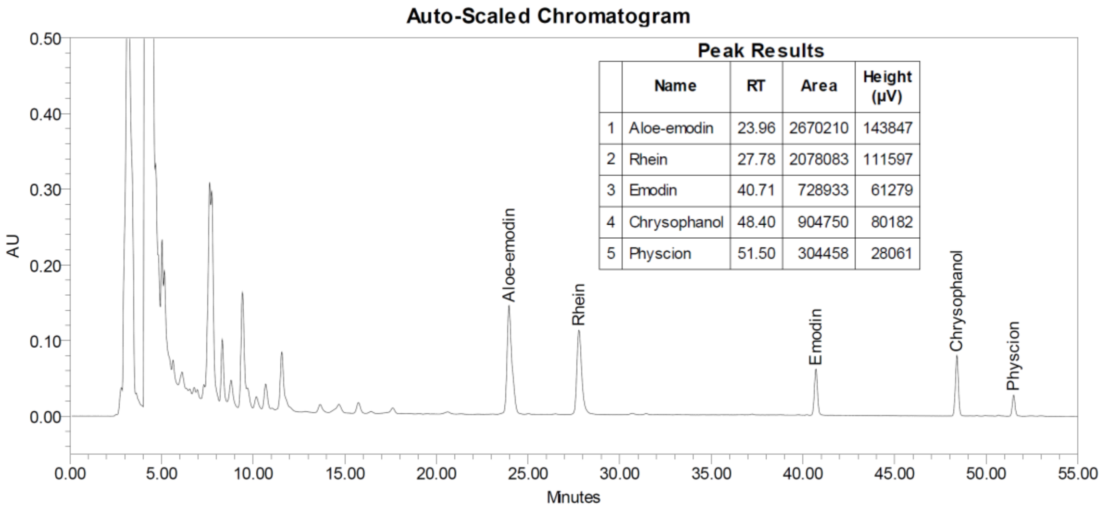

(b) Qualitative analysis for Sennoside A and Sennoside B detection

HPLC conditions

Detection wavelength	270 nM
Flow rate	1.2 mL/min
Run time	80 min

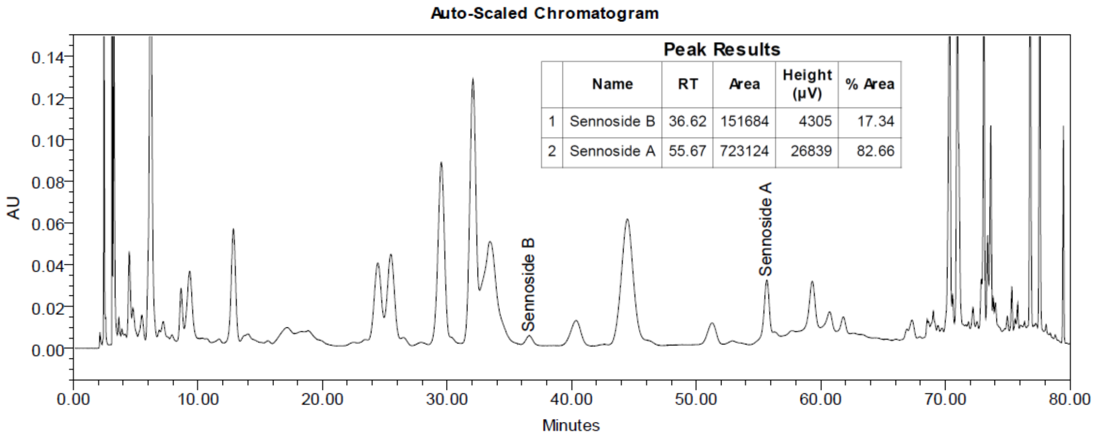

(c) Quantitative results

Marker compound in Rex	quantity (mg/g)
**Sennoside A**	**10.19**
**Sennoside B**	**1.4**
**Aloe-emodin**	**13.8**
**Rhein**	**3.58**
**Emodin**	**1.91**
**Chrysophanol**	**2.61**
**Physcion**	**0.65**

Supplementary Figure S4. HPLC fingerprinting and quantitation of Rex. A Cosmosil 5C18-MS-II column was used as the stationary phase, and a gradient of 0.085% phosphoric acid, acetonitrile, and water as the elute solution. The UV detection wavelength was set at 270 nm. The analytical run time was 80 min for sennoside A and sennoside B detection. The analytical run time was 55 min for aloe-emodin, rhein, emodin, chrysophanol, and physcion detection. Qualitative (a-b) and quantitative (c) data were showed.
